# Advancements in Understanding Gastric Cancer: A Comprehensive Review

**DOI:** 10.7759/cureus.46046

**Published:** 2023-09-27

**Authors:** Khizer K Ansari, Vasant Wagh, Azeem I Saifi, Iram Saifi, Sharad Chaurasia

**Affiliations:** 1 Medicine and Surgery, Jawaharlal Nehru Medical College, Datta Meghe Institute of Higher Education & Research, Wardha, IND; 2 Community Medicine, Jawaharlal Nehru Medical College, Datta Meghe Institute of Higher Education & Research, Wardha, IND; 3 Microbiology, Jawaharlal Nehru Medical College, Datta Meghe Institute of Higher Education & Research, Wardha, IND; 4 Radiology, Jawaharlal Nehru Medical College, Datta Meghe Institute of Higher Education & Research, Wardha, IND

**Keywords:** advance treatment, ai, prevention and screening, biomarkers, environmental and demographic risk factors, gastric cancer

## Abstract

As a complex and difficult condition, gastric cancer (GC) continues to have a big impact on the world's health. The goal of this review article is to give a thorough summary of the most recent developments and research discoveries in the field of stomach cancer. The review discusses a wide range of topics, such as the epidemiology and risk factors for GC, molecular insights into its pathogenesis, the use of biomarkers in diagnosis and prognosis, current and novel therapeutic approaches, and the intriguing potential of immunotherapy. In addition, procedures for surgery, therapy strategies, and imaging modalities for diagnosis and staging are examined. The paper emphasizes how crucial it is to comprehend the tumor microenvironment and how it affects the course of the disease. Overall, this review provides a comprehensive assessment of the current body of knowledge, highlights research gaps, and suggests future lines of inquiry to enhance the treatment of GC.

## Introduction and background

In the past, both in the United States and internationally, gastric cancer (GC) held the title of the most common cancer [1]. However, over time, other cancer types have surpassed it in terms of overall incidence. Despite this, GC remains the second leading cause of deaths globally and continues to significantly contribute to cancer-related deaths. Moreover, it remains the most prevalent form of cancer in Eastern Asia [1].

This article reviews both traditional studies and recent data that have contributed to the body of literature in the last several years, addressing the epidemiology and prevention of GC. The bulk of GCs, about 90% of them, are adenocarcinomas that develop from the mucosa or superficial layer of the stomach glands. Therefore, unless otherwise stated, the main subject of our discussion is adenocarcinomas. However, there are other types of stomach cancer, such as leiomyosarcomas and mucosa-associated lymphoid tissue (MALT) lymphomas, which develop from the muscles surrounding the mucosa and come from lymphoid tissues.

According to Lauren's classification, the two basic histological kinds of GC adenocarcinomas are diffuse and intestinal [2]. These two groups not only differ in how they look under a microscope but also in terms of the gender ratio, age at diagnosis, and other epidemiologic traits [3]. One of the anatomical subsites of the stomach is the cardia, which is about an inch long and situated at the top of the stomach. Other anatomical subsites include the fundus, body, pylorus, and antrum. These subsites might be identified by their different histologies, anatomical divisions, or both.

For the purposes of this inquiry, it is especially important to distinguish between adenocarcinomas that originate from the cardia (known as cardia GC) and those that originate from other areas of the stomach (known as non-cardia GC). As these two groups have different epidemiological patterns and etiological factors, they must be differentiated from each other.

## Review

Methodology

**Figure 1:**
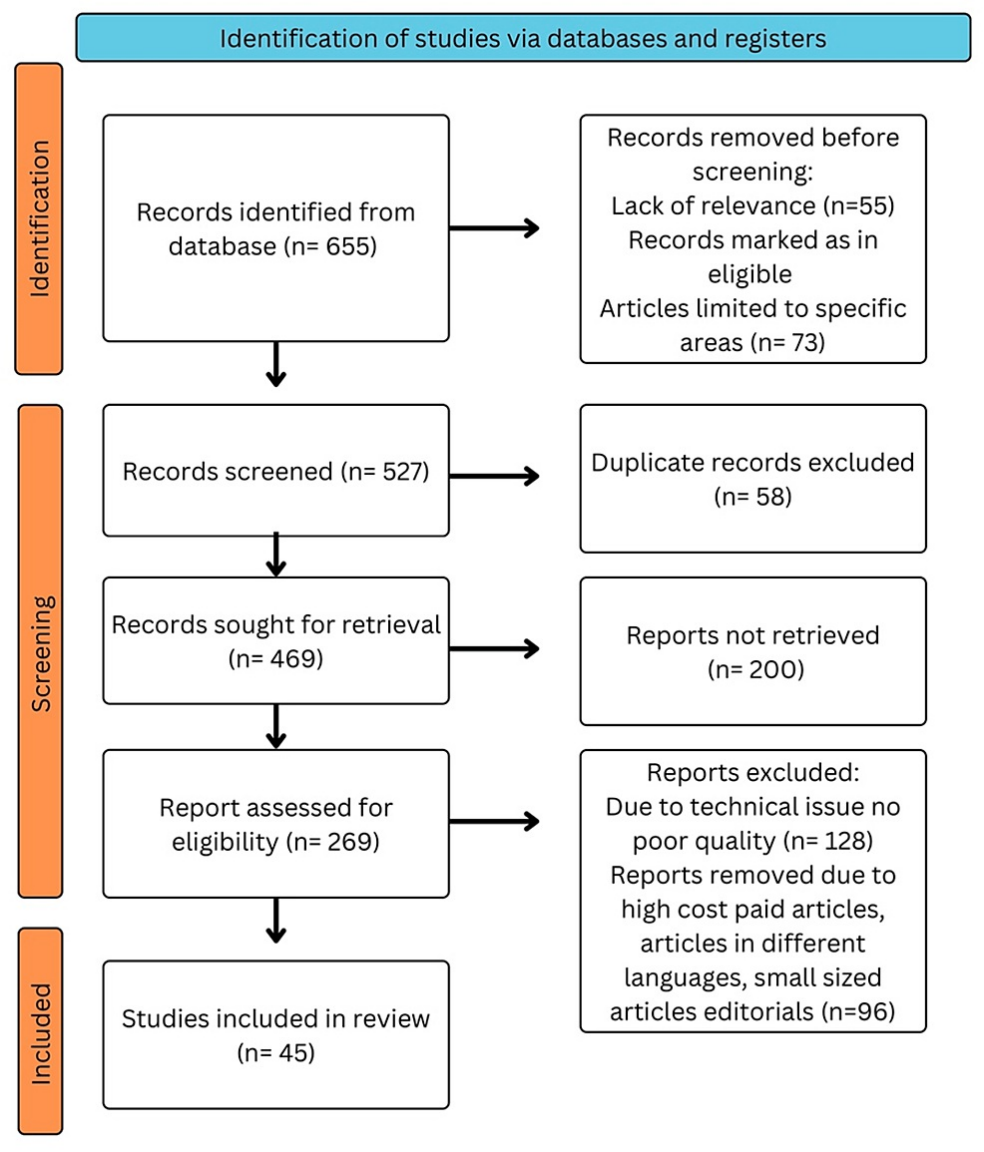
PRISMA methodology PRISMA: Preferred Reporting Items for Systematic Reviews and Meta-Analyses Image credits: Khizer K. Ansari

We performed a comprehensive search in the electronic databases PubMed, MEDLINE, Embase, Google Scholar, and ResearchGate, and a search of the English-language literature was done. It was also the subject of a different search. The query terms were "Advance treatment " OR "Modern therapy"; "Gastric cancer " OR "Gastric carcinoma; "development," OR "progression”; “Medical management of gastric cancer” OR “Gastric malignancy intervention”; "Artificial intelligence ” OR "Prevention and screening.” The articles in this review meet the following requirements: studies conducted exclusively on advancements in understanding GC and new treatment interventions and studies conducted in English over the preceding 10 years. Figure 1 highlights the Preferred Reporting Items for Systematic Reviews and Meta-Analyses (PRISMA) method's use in the research methodology.

Trends

The incidence rates of GC have decreased in the majority of locations [4,5]. Between 1992 and 2010 in the United States, rates fell by 1.7% for men and 0.8% for women per year [6]. In contrast to this downward trend, cardia GC rates have remained stable or even increased in Western countries [7,8]. Different reasons may lead to distinct developments in cardiac and non-cardia GC. Unlike non-cardia GC, *Helicobacter pylori* is not a risk factor for cardia GC in Western countries [9], which influences cardia GC rates differently. The growing incidence of obesity in Western countries [10] makes obesity and gastroesophageal reflux appear to be risk factors for coronary GC. The apparent increase in gastric cardia adenocarcinoma rates may also be attributed to improvements in the categorization of stomach tumors [11]. Recent outliers, such as the rise in GC among the young White populations in the United States [12], warrant further study for confirmation. Understanding these patterns and causes is crucial for developing specialized preventative and treatment methods [7,12].

Environmental and demographic risk factors

**Figure 2:**
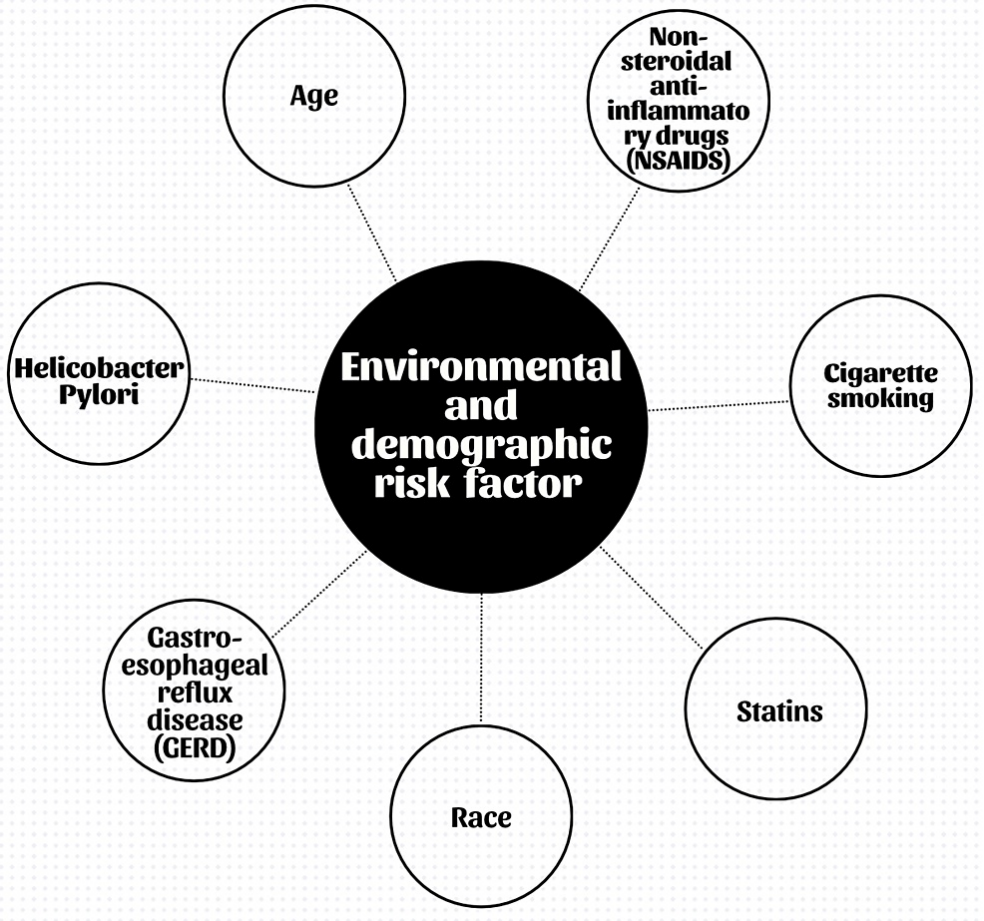
Various environmental and demographic risk factors Image credits: Khizer K. Ansari

Numerous inherited and environmental variables contribute to the development of GC. While certain risk factors, such as age and sex, cannot be changed, others, such as smoking and *H. pylori* infection, may. Importantly, cardia and non-cardia GC may be affected differently by risk variables. Age, male gender, radiation exposure, cigarette usage, and family history are typical risk factors for both [13]. The use of aspirin and statins has the ability to stop both types of GC. Race also has an impact; in the United States, White people are more likely to have cardiac GC, while Hispanic people are more likely to have non-cardia GC. Cardia GC is specifically linked to gastroesophageal reflux disease (GERD) and obesity, unlike non-cardia GC. By contrast, there are specific risk factors for non-cardia GC, including *H. pylori* infection (especially in Western nations), poor socioeconomic level [13], and perhaps dietary variables, such as insufficient fruit and vegetable consumption and excessive consumption of salty and smoked foods. Understanding these unique risk factors is crucial for individualized care and prevention of various GC types (Figure 2).

Age

The risk of GC gradually increases with age. In the United States, between 2005 and 2009, 1% of GC cases were identified in people aged 20 to 34 years, while 29% of cases were reported in people aged 75 to 84 years. The median age at diagnosis during this period was 70 years [14].

Cigarette Smoking

The International Agency for Research on Cancer (IARC) reaffirmed the association between smoking and GC in 2002 [15]. According to a meta-analysis, male smokers had a 60% greater risk of GC than non-smokers (respiratory rate (RR): 1.6), whereas female smokers had a 20% higher risk (RR: 1.2) [16]. Smokers who have quit had a lower association. Smoking increases the chance of developing cardiac and non-cardia GC, albeit specific research findings may differ [15-18]. Smoking hookah has also been linked to an increased risk of GC [19]. There is a need for more study to corroborate these correlations because not all studies consistently demonstrate them [20].

Race

GC among White people appears to be about twice as common as in other racial groups (26), although non-cardia GC is about half as common. Asians and Pacific Islanders have the highest chance of developing non-cardia GC in the United States, followed by Black and Hispanic, with White people having the lowest incidence [7]. Environmental rather than genetic variables appear to have a greater influence on race-related differences in GC occurrence. Notably, Japan has one of the highest GC incidence rates worldwide [4]. When Japanese people immigrate to the United States, their GC rates remain elevated in the first generation before declining and becoming comparable to those of Americans of European ancestry in future generations [21].

Helicobacter pylori (H. pylori)

The discoverers of *H. pylori* cleverly hypothesised that the organism may have a role in poorly understood gastritis-related disorders, such as peptic ulcer and stomach cancer, in their landmark publication in the *Lancet* [22]. With a relative risk of about six for non-cardia GC, extensive research carried out over the previous 20 years has established *H. pylori* as a certain cause of stomach cancer [23]. GC risk has been associated with specific *H. pylori* strains, particularly those that carry the CagA virulence factor [23-25]. According to estimates, *H. pylori* causes somewhere between 65% and 80% of instances of GC or over 660,000 new cases every year [23,26]. However, given the probable underestimating of prevalence in prior research that used the enzyme-linked immunoassay (ELISA) detection method, these figures may be conservative. Western blot assay-based recent studies suggest a relative risk closer to 21 [27]. In Western nations, *H. pylori* is notably associated with a higher incidence of non-cardia GC, but not as strongly with cardia GC [24]. It is thought that the lowering prevalence of *H. pylori*, probably brought on by better hygiene habits and widespread antibiotic usage, is a factor in the declining incidence of non-cardia stomach cancer [28,29]. Although the specific processes by which *H. pylori* causes GC are still not fully understood, two putative pathways, namely, direct impacts on epithelial cells and indirect effects involving inflammation in gastric epithelial cells, are frequently mentioned. In addition, *H. pylori* may alter the function of epithelial cells through bacterial substances, such as CagA . Although the interactions between these pathways are not fully understood, it is believed that they work in concert to favor the growth of GC [30]. 

Nonsteroidal Anti-Inflammatory Drugs (NSAIDS)

Aspirin and other nonsteroidal anti-inflammatory medicines (NSAIDs) may reduce the chance of developing GC, according to recent research. Such relationships have been observed by several meta-analyses of observational studies, which demonstrate a reduced incidence of both cardia and non-cardia GC with NSAID use. A study that found an inverse correlation with non-cardia GC but not cardia GC illustrates the fact that there are some differences. Aspirin use was found to be generally inversely associated with all forms of GC in the most recent meta-analyses, which included 13 and 15 research, respectively, with similar findings in case-control and cohort studies. These examinations, however, did not produce any specific findings for the detailed GC sub-sites. Given that this study was based on a small number of GC fatalities, it is intriguing that a pooled analysis of seven clinical studies looking into the use of daily aspirin for the prevention of vascular events did not show a decreased risk of GC-related death in comparison to the control group [31].

Statins

According to two recent meta-analyses, taking statins may reduce the incidence of GC by as much as 30%. However, the risk was reduced by about 15% when a research with outlier results was eliminated, and the outcomes were similar across trials. Other malignancies, such as esophageal adenocarcinoma, have also been related with statin use having a lower chance of development, and a number of mechanisms have been postulated to account for this. Statins are not consistently linked to a lower risk of all cancer incidence or mortality, especially in randomized studies, which is crucial to remember. Therefore, more research is required to fully comprehend the potential connection between statin use and a lower risk of GC [31].

GERD

With a five- to sevenfold higher risk, GERD is firmly linked to an elevated risk of esophageal adenocarcinoma. Numerous research works have also discovered strong links between GERD and GC, with risk increases of two- to fourfold in the majority of investigations, but other studies have produced contradictory findings. One type of cardia GC, comparable to esophageal adenocarcinoma linked to GERD, and another form, similar to non-cardia GC linked to severe atrophic gastritis and *H. pylori *infection, have been proposed as two separate forms of the condition. If this pattern is accurate, it might help to explain why some groups do not show any association. Similar to the relationship between GERD and esophageal adenocarcinoma, where GERD can result in columnar and intestinal metaplasia (IM) that may proceed to adenocarcinoma, the mechanism underlying the linkage between GERD and cardia GC may be similar. However, it is crucial to keep in mind that because of their proximity and overlapping location, esophageal adenocarcinomas might occasionally be mistaken for cardiac malignancies. Meanwhile, the relationship between atrophic gastritis and non-cardia GC may help to explain the null or inverse association that has been found between GERD and non-cardia GC. GERD risk is decreased, and stomach acid output is decreased in severe atrophic gastritis [31].

Prevention

Primary and Secondary Prevention

GC prevention's ideal and ultimate goal is to reduce cancer incidence and mortality rates. Both primary and secondary preventative techniques are used in GC prophylaxis. Avoiding recognized carcinogens, boosting the host defence mechanisms, altering one's lifestyle, and chemoprevention are all parts of primary prevention [32]. Eliminating the offending pathogen must be taken into consideration as a primary preventative strategy in malignancies caused by infections [33]. Secondary prevention includes detecting and treating premalignant lesions or early-stage cancers [34]. The latter can alternatively be viewed as monitoring those whose sickness has been established or tertiary prevention. The most effective way to prevent cancer employs both a medical strategy and epidemiological strategy. By improving lifestyle, removing risk factors, and complementing it with anti-carcinogenic preventive measures, the epidemiological method seeks to lower cancer incidence and death. By administering medications with direct anticarcinogenic activities, the medical approach aims to eliminate the responsible germ and prevent the growth of cancer. It has been examined for the chemoprevention of GC to eradicate *H. pylori* using an antibacterial therapy in conjunction to the administration of NSAIDs, such as aspirin [32].

Insights and Implications From Research on the Evaluation of H. pylori Eradication in People With a Family History of GC

A family history of the illness is a recognized risk factor for stomach cancer, which is influenced by a variety of factors. Similar factors, such as inherited factors and early environmental factors, have been related to an increased risk of GC in studies of relatives of patients. According to studies examining *H. pylori* infection and gastric mucosal alterations in family members, the first-degree relatives of GC patients have a higher prevalence of *H. pylori* infection, as well as more advanced stages of mucosal atrophy and IM, compared to control groups. Studies focusing on young relatives of GC patients identified before the age of 40 have similarly revealed greater rates of *H. pylori* infection and advanced stages of IM in the stomach corpus mucosa. Similar research done in Western nations has shown that the first-degree relatives of GC patients are more likely to develop *H. pylori* infection, progressive gastric mucosal atrophy, and IM, even in young children. Patients with GC in their families are usually encouraged to have *H. pylori* removed by current recommendations, despite the fact that there is no conclusive evidence that the prevalence of GC has greatly decreased in this population in particular. The effects of *H. pylori* eradication on inflammation, atrophy, and IM in the first-degree relatives of GC patients were investigated in a study by Massarrat et al. While stomach atrophy decreased after eradication, IM did not, even in its early phases, demonstrate any discernible improvement. In addition, a follow-up period of more than four years revealed that untreated cases of *H. pylori* infection progressed more quickly than *H. pylori*-eradicated cases in terms of gastric atrophy and IM in the antrum [35].

Strategies for the Prevention and Surveillance of Metachronous Cancer Following Endoscopic Resection

Metastatic GC cases are regularly seen in various locations of the stomach mucosa after the endoscopic excision of the primary tumor. According to a multicenter research on metachronous stomach tumors following endoscopic resection, getting rid of *H. pylori* dramatically lowers the chance of developing new GC, especially in people who are already at high risk. This study also discovered that patients with IM and mucosal atrophy benefit from getting rid of *H. pylori*. However, other investigations have revealed that the only people for whom the removal of *H. pylori* has a protective effect on the risk of stomach cancer are those without IM or gastric mucosal atrophy. The group that still harbored *H. pylori* had a higher risk of cancers than the group that had the virus eradicated in a retrospective examination of people with early GC who underwent endoscopic resection and developed metachronous GC. According to a separate study by Kato et al., effective *H. pylori* eradication lowers the risk of patients developing metachronous stomach cancer following endoscopic resection and significantly lowers cancer rates in the eradicated group compared to the control group. The elimination of *H. pylori* efficiently slows the progression of stomach mucosal lesions in the precancerous stage, according to several studies. The eradication of *H. pylori* infection may have the ability to completely suppress latent tumors, including those too tiny to be seen by endoscopy, while also postponing tumor growth. *H. pylori* infection is linked to the development and progression of stomach cancer [35].

H. pylori Eradication for GC Prevention

The IARC has designated *H. pylori* as a class I carcinogen due to compelling data connecting it to the emergence of GC. *H. pylori*'s function as a risk factor is significantly influenced by the virulence factors, which are especially expressed by cagA strains. The primary factors contributing to gastritis, which causes stomach mucosal inflammation, oxidative damage, and DNA nitrosation, are involved in *H. pylori*-induced carcinogenesis. Following what is known as Correa's cascade, this chronic inflammation develops into atrophy, IM, dysplasia, and ultimately malignancy. The likelihood of GC is directly correlated with the degree and severity of IM and stomach atrophy. The cumulative incidence of cancer at various times following infection discovery was published in a recent large retrospective cohort research conducted in the United States comprising over 370,000 patients with *H. pylori* infection. At ages 5, 10, and 20, the study found incidence rates of 0.37%, 0.5%, and 0.65%, respectively. Minorities of all races and ethnicities, as well as smokers, were shown to have a much-increased chance of developing GC. Moreover, the study showed that treatment of *H. pylori* infection decreased risk only if eradication was successful [36]. The most recent meta-analysis, conducted by Lee et al. in 2016, found that eliminating* H. pylori* reduced GC risk by around 35% [37,38]. This benefit was more significant in patients without IM, where the risk was reduced by about 75%. Given that *H. pylori* eradication therapy appears to significantly lower the risk of GC in first-degree relatives, it should also be made available to people who already have the infection. The elimination of *H. pylori* also decreased the incidence of metachronous GC, which in turn reduced stomach atrophy, in patients with early GC, according to a recent trial [39]. Therefore, *H. pylori* eradication is advised by worldwide guidelines as the best method for the primary prevention of GC, particularly in people without IM [40].

Screening

General Guidelines for Cancer Screening in the Context of an Organized Program

The decrease in mortality from a particular cancer can be used to assess the success of a population-based cancer screening program. The degree of organization and the effectiveness with which various screening process components are used determine the success of a screening program. For the World Health Organization (WHO), Wilson and Jungner set the criteria for illness screening in 1968 [41]. The accuracy of the screening test method, illness management, epidemiology, and cost effectiveness are only a few of the considerations made in these criteria. A screening test's high sensitivity is an important feature that makes sure that cases of the disease are not missed when they are still treatable. The IARC has identified the qualities that such a program should have to satisfy the essential standards. Organized cancer screening is thought to be the most effective strategy for reaching the desired goals [42].

Current Nationwide Screening Programs

GC screening programs have been put in place statewide in Japan and South Korea. The screening program was started in Japan in 1960, and the preferred screening technique is photofluorography following a barium meal. However, despite the fact that funding for *H. pylori* eradication therapy was authorized in February 2013, there is still no formal screening program for this condition in Japan. The screening program in South Korea combines upper endoscopy and photofluorography screening. This method improves population-wide stomach cancer detection. In addition, Kazakhstan has decided to implement a bi-annual upper endoscopy screening program for esophageal and stomach malignancies. The program, which has been in place since the beginning of 2013 in six of the 16 regions of the nation, intends to be implemented throughout the entire nation. The program's structure, however, might not entirely meet the requirements for an organized screening program, which could limit its ability to accomplish the necessary aims (Table 1) [35].

**Table 1:** Various gastric screening programs GC: gastric cancer Reference: [35]

Country	Screening program	Screening technique	Implementation details
Japan	Statewide GC screening since 1960; no formal screening for *H. pylori* despite funding for therapy.	Photofluorography after barium meal	GC screening started in 1960. No *H. pylori* screening despite authorized funding.
South Korea	Statewide GC screening combining upper endoscopy and photofluorography	Upper endoscopy and photofluorography	Combination of upper endoscopy and photofluorography for enhanced stomach cancer detection
Kazakhstan	Bi-annual upper endoscopy screening for esophageal and stomach malignancies	Upper endoscopy	Started in 2013 in six regions, planned for nationwide implementation. Structure might not fully align with organized screening standards, potentially limiting effectiveness in achieving goals.

Currently Available Non-Invasive Testing

**Figure 3:**
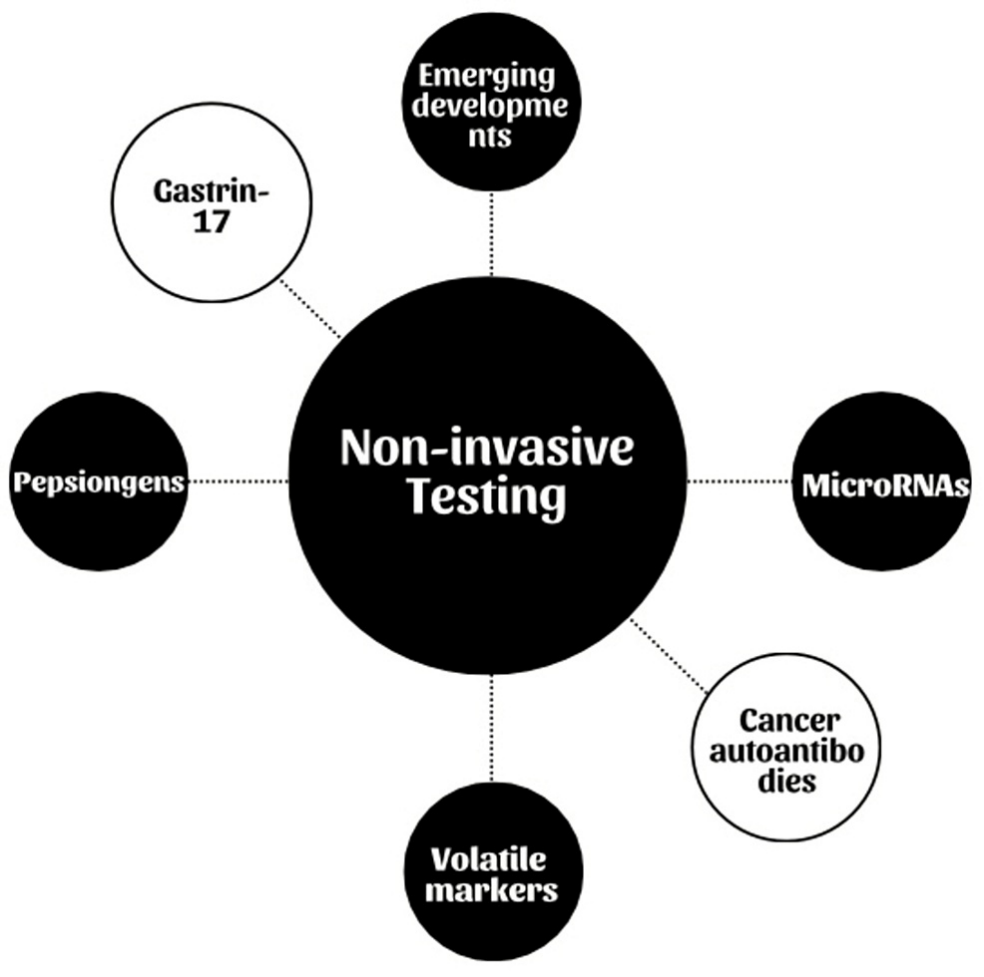
Various non-Invasive esting Image credits: Khizer K. Ansari

Due to the unique epidemiology of stomach cancer and financial restrictions, the broad use of endoscopy or photofluorography screening methods as population-based gastric screening tools seems unlikely outside of Asia. Therefore, it becomes crucial to carefully examine the options and factors related to the adoption of non-invasive screening techniques (Figure 3).

Pepsinogens

Pepsinogens are digestive enzymes whose blood levels can serve as a proxy for an understanding of how well the stomach is working. In contrast to pepsinogen II (PgII), which is produced by a variety of stomach cells, pepsinogen I (PgI) is produced by certain cells in the stomach. Although only a few of these enzymes actually enter the bloodstream, it is still enough to evaluate stomach function. Pepsinogen levels rise during inflammation but fall during atrophic gastritis. The ratio of PgI to PgII (PgI/II) is thought to be a more accurate predictor than PgI alone, particularly when atrophy and *H. pylori* infection combine. The diagnostic cut-off values for PgI and PgI/II, however, differ between investigations. Absolute values differ across Asia and Europe due to the usage of various test systems and methodologies. As a result, locally verified test systems are advised. While pepsinogen testing has demonstrated good sensitivity for detecting atrophy in screening settings, it has performed less satisfactorily in diagnosing GC, potentially missing a sizable portion of GC patients. As a result, prior to incorporating these tests, particularly outside of Asia, in structured screening programs, regional validation and pilot studies are required [35].

Gastrin-17

Amidated gastrin-17 (G-17), a different marker for measuring atrophy in the stomach's antral region, has been proposed. The GastroPanel test is used to evaluate *H. pylori* IgG antibodies, G-17, and pepsinogens (PgI and PgII) in Europe. The actual results of this test, however, fall short of expectations. Medication, food intake, and inflammation are just a few of the variables that have an impact on G-17 levels in plasma. The ideal way to assess how well antral G-cells are working is to measure G-17 levels after a protein-rich meal, but this is impractical for screening. Numerous studies evaluate fasting G-17 levels, but the test's sensitivity during a fast or after food stimulation (15.8% during a fast and 36.8% after stimulation) is deemed insufficient for screening purposes. Although it is claimed that the GastroPanel test, which includes G-17, may detect gastric mucosal atrophy with accuracy, this performance appears to depend more on the pepsinogen tests than just G-17 by itself [35].

Emerging Developments

Using molecular biology techniques to identify the risk of GC has drawn more attention in recent years. It is vital to emphasize that this study will not cover hereditary GC instances linked to CDH1 mutations because there are currently management guidelines for people who are at risk. There is currently no widely accepted polymorphism of proinflammatory cytokines for individual patient risk stratification, despite the fact that substantial research has been done on the function of host genetics in GC risk stratification. This is mostly caused by the absence of reliable screening techniques and associations in this situation [35].

MicroRNAs

Small RNA molecules called microRNAs control the expression of genes. Due to their stability in multiple tissues, they have the potential to be useful diagnostic and prognostic indicators for a variety of malignancies, including GC. To pinpoint particular microRNA signatures linked to GC and its precursor lesions, extensive studies have been done. Although there have been numerous publications on this subject, further research is required to identify a trustworthy microRNA signature for the early detection of GC and to evaluate the reproducibility of results across various groups [35].

Cancer Autoantibodies

A promising method for the early diagnosis of GC is a panel of particular cancer autoantibodies. In numerous cancer forms, autoantibodies against antigens linked with tumors have been found. Although it may be difficult to find antibodies that specifically target tumor-associated antigens, researchers are now adopting a panel-testing strategy to look into cancer-specific antibodies. A 45-autoantibody profile in GC has been found to have a 59% sensitivity and 90% specificity for separating GC from healthy controls. This shows that the panel-testing approach may be able to help with the early diagnosis of GC [35].

Volatile Markers

Volatile substances found in exhaled breath have the potential to be an accurate and simple way to identify cancer. These volatile indicators can be located using gas chromatography in conjunction with mass spectrometry or nanosensor technology. A recent pilot study showed that it is possible to distinguish between those with benign gastrointestinal diseases and those who have stomach cancer using a highly sensitive, cross-reactive, nanomaterial-based gas sensor. The sensor detected GC using volatile marker patterns and attained a sensitivity of 89%, a specificity of 90%, and an accuracy of 90%. It is vital to keep in mind, nevertheless, that regional variations in the makeup of volatile compounds may call for local adaptation of the procedure through "teaching of the electronic nose" [35].

Developing serum biomarker panels for accurate GC diagnosis: unveiling the diagnostic potential

Due to its simple accessibility and non-invasive collection, serum is a useful tool for clinical diagnostics. There is a need for more clinically applicable biomarkers because the blood biomarkers for GC that are currently available lack specificity and sensitivity. This work is the first to use antibody array techniques to pinpoint serum biomarkers for GC, screening serum biomarkers with increased clinical specificity and sensitivity utilizing a high-throughput solid antibody array. Eleven cytokines (IFNGR1, Notch-3, TNFRSF19L, GHR, SLAMF8, FR-beta, integrin alpha-5, galectin-8, EphA1, epiregulin, and FGF-12) that were significantly higher in GC serum relative to controls were found using the antibody array used in the study. The Mann-Whitney U test was used for the analysis. The microarray data's unsupervised hierarchical cluster analysis also successfully distinguished between the GC and control groups, adding more information about how cytokines are expressed differently in both groups.

Fresh samples were used in the study's enzyme-linked immunosorbent test (ELISA) to corroborate the results, which showed that several cytokines had undergone significant alterations that the antibody array had detected. This implies that these cytokines increase in GC serum with consistency and may act as serum indicators for the illness. Notably, IFNGR1, a crucial component of the IFN-signaling system, was shown to be enhanced in GC serum, and earlier research has connected an IFNGR1 gene polymorphism with a higher chance of developing early GC. For the first time, in this investigation, higher levels of other cytokines, including Notch-3, TNFRSF19L, GHR, SLAMF8, FR-beta, and integrin alpha-5, were seen in GC serum. For instance, Notch-3, a member of the Notch receptor family, is recognized as being overexpressed in stomach and intestinal carcinomas. Additionally discovered to be increased in GC was TNFRSF19L, a brand-new member of the tumour necrosis factor receptor superfamily, suggesting a potential function in the growth of cancer.

Prior to this study, there was no connection between GC and GHR, a member of the class I cytokine receptor family that has been related to a number of malignancies. The enhanced expression of SLAMF8, FR-beta, and integrin alpha-5 in GC serum also revealed new information about their probable involvement in GC. Galectin-8, EphA1, epiregulin, and FGF-12 were also discovered to be raised in GC serum in this investigation. These substances have previously been documented to be upregulated in GC. Six of these 11 cytokines (IFNGR1, TNFRSF19L, GHR, SLAMF8, FR-beta, and integrin alpha-5) have the potential to be novel serum biomarkers for the early detection and prognosis of GC, according to the findings, which collectively imply that these 11 cytokines may have a role in the occurrence and progression of GC. As a result, a panel of 11 cytokines that were significantly increased in GC serum were found by this investigation. These results offer light on the function of these cytokines in the disease and emphasise their potential as blood biomarkers for GC. To explore and validate the diagnostic and prognostic value of these biomarkers in broader populations, additional study is required [43].

Adverse reactions of radiotherapy

Radiation-induced gastrointestinal injury is still a problem even if advances in radiotherapy technology have reduced the amount and dose of radiation given to the stomach. Gastroscopy is necessary for patients who have symptoms, such as anorexia, indigestion, a burning feeling, nausea, vomiting, upper abdominal discomfort, bleeding, or perforation. Due to its limited radiation resistance, the small intestine can potentially be harmed by radiation. Dehydration, electrolyte imbalances, infection, hemorrhage, and even death can result from acute intestine mucosal constriction, edema, and even stripping. Some patients may continue to experience decreased quality of life even though the majority of patients' quality of life returns to baseline within 6-12 months after radiation.

Treatment stoppage or dose decrease during acute responses should be taken into consideration to enhance the patient's quality of life. In individuals with stomach cancer, perioperative radiation significantly lowers the rate of local recurrence and increases survival. It can be helpful for people who are unable to perform a D2 radical resection with a negative margin because it raises the likelihood of a successful surgical procedure or total tumor eradication. Due to a dearth of heart-related randomized controlled trials, the value of preoperative irradiation must yet be investigated. Patients with D1 and D2 disease, particularly those with stage III disease, require postoperative irradiation. Palliative radiation can also improve curative outcomes and lessen side effects from the treatment when used for advanced stomach cancer. Future studies should also concentrate on selecting the right patient populations for combined treatments for stomach cancer and screening novel, individually tailored radiosensitivity markers [44].

Precision surgical treatment of GC

As surgery allows total elimination of the disease, it is the main treatment for stomach cancer. There are continual discussions about the best surgical practices in the field of stomach cancer surgery, which is a topic that is constantly developing. However, a global consensus on these issues is progressively emerging owing to the participation of professionals and the release of reliable studies. As GC is a highly diverse tumor, several subtypes display different biological characteristics. GC develops via several molecular pathways, including cell proliferation, metastasis, and the epithelial-mesenchymal transition (EMT). With the development of molecular biology technologies, personalized molecular and immunotherapy, as well as molecular typing, have become possible treatment modalities for GC. The advancement of preoperative imaging assessment, the use of surgical robots and minimally invasive techniques, the use of intraoperative fluorescent navigation for precise operations, and the implementation of sophisticated perioperative management based on the concept of accelerated rehabilitation are the directions of GC treatment in the era of precision medicine [45].

Revolutionizing GC surgery: harnessing artificial intelligence and big data for precision

The 1956 Dartmouth College meeting and early research in the 1970s are where the history of artificial intelligence (AI) in medicine begins. A branch of AI called "deep learning" has shown substantial growth and shows potential for real-world use. Integration with other medical systems, such as electronic medical records, outcome reporting, and genomic/proteomic data processing tools, is essential to maximizing its potential. Drug discovery, disease detection, and treatment have all benefited greatly from AI. In countries with a high incidence of stomach cancer but low detection rates for early-stage cases, for instance, a computer-aided method showed great accuracy in diagnosing early GC. Computer-assisted methods for early diagnosis could be useful for GC, a serious public health issue in China. Pathology is a crucial area for AI use, especially in stomach cancer surgery, and includes staining analysis, early cancer screening, benign and malignant tumor diagnosis, and tailored medication development.

In surgical pathology, automatic image analysis is a prominent issue. Standardized diagnosis and therapy are key components of precision medicine in GC surgery, and big data and AI play critical roles in the gathering, processing, and use of data. However, the lack of unifying standards in the current information systems makes it difficult to conduct studies with little data and different treatment approaches, which reduces generalizability and costs money. A comprehensive big data research platform for gastrointestinal oncology is being developed with the goal of advancing standardization, enhancing data quality, and facilitating collaboration. It is vital to remember that AI cannot fully reproduce some parts of medical treatment, such as the human-emotional bond between doctors and patients. Large database construction also prompts worries regarding patient privacy and data security [45].

## Conclusions

This in-depth review paper illuminates the complex and quickly changing field of research and treatment for stomach cancer. Key risk variables have been identified through the study of epidemiological data, which may aid in the creation of preventive measures and awareness-building campaigns. Targeted therapy and personalized medicine strategies have been made possible by molecular insights into the pathophysiology of GC, demonstrating encouraging results for individuals with certain genetic abnormalities. In addition, the potential of biomarkers to aid in early detection and forecast treatment results emphasizes the significance of precision medicine in the management of GC. The study also emphasizes the value of immunotherapy as a viable strategy for treating advanced GC, giving patients with few therapeutic choices fresh hope.

The investigation of various imaging methods, surgical procedures, and therapeutic philosophies also highlights the necessity of multidisciplinary cooperation to maximize patient care.Though there have been advancements in our understanding of stomach cancer, there are still a number of problems. To fully understand the intricacies of the tumor microenvironment and the underlying processes of treatment resistance, more study is necessary. Large-scale clinical trials are also required to create standardized treatment regimens and confirm the safety and effectiveness of novel medicines. Overall, this study is an important resource for cancer researchers, doctors, and decision-makers. It highlights the significance of ongoing research initiatives, knowledge exchange, and teamwork to deepen our comprehension of stomach cancer and eventually increase patient outcomes. Together, we can work to overcome these obstacles in order to progress the detection, diagnosis, and treatment of stomach cancer, paving the way for a better future for patients and their families.
